# Educational Effectiveness of a 5-Country Virtual Exchange Program for Internationalization in Occupational Therapy Education: Mixed Methods Study

**DOI:** 10.2196/77564

**Published:** 2025-11-06

**Authors:** Natsuka Suyama, Kaoru Inoue, Norikazu Kobayashi, Anuchart Kaunnil, Supatida Sorasak Siangchin, Muhammad Hidayat Sahid, Erayanti Saloko, Sk Moniruzzaman

**Affiliations:** 1 Department of Occupational Therapy, Graduate School of Human Health Sciences, Tokyo Metropolitan University, 7-2-10 Higashiogu, Arakawa-ku, Tokyo, 1168551, Japan, 81 3-3819-1211; 2 Department of Occupational Therapy, Faculty of Associated Medical Sciences, Chiang Mai University, Chiang Mai, Thailand; 3 Division of Occupational Therapy, Faculty of Physical Therapy, Mahidol University, Nakhon Pathom, Thailand; 4 Occupational Therapy Study Program, Department of Applied Health, Vocational Education Program, University of Indonesia, Depok, Indonesia; 5 Department of Occupational Therapy, The Health Polytechnic of Surakarta, Surakarta, Indonesia; 6 Department of Occupational Therapy, Bangladesh Health Professions Institute, Dhaka, Bangladesh

**Keywords:** virtual exchange program, occupational therapy education, global education, international orientation, intercultural competence, health professions education

## Abstract

**Background:**

Global health care education that cultivates international orientation is important for providing medical care in consideration of diverse backgrounds and collaboration with foreign medical professionals. Virtual international exchange programs could be a new type of global education in the present postpandemic era.

**Objective:**

This study aimed to examine the effectiveness of a virtual international exchange program in fostering quality academic and professional learning and international orientation from student perspectives across 5 countries. This research is expected to contribute to education for the development of global human resources in the health professions.

**Methods:**

This quasi-experimental study used a before-and-after design using a convergent parallel mixed methods approach. In this study, a 5-day interactive virtual program was offered to occupational therapy students from Bangladesh, Indonesia, Japan, the Philippines, and Thailand. The students were asked about their expectations and international orientation before the program, and about their evaluation of the program and international orientation afterward. Numerical data from a questionnaire on program expectations and evaluations were analyzed using descriptive statistics. Data on international orientation were subjected to qualitative analysis using steps for coding and theorization.

**Results:**

In total, 29 students participated in the program, out of which 12 students (response ratio 41.4%) answered the research questionnaires both before and after the program. Overall, the students’ expectations of the program were met in terms of expertise, scientific learning skills, and group interactions. Comparing before and after the program, mean scores of how the program met expectations increased, and the mean scores after the program in all 12 items asking about program evaluation were from 3.8 (SD 1.19) to 4.9 (SD 0.67; range: score 1 [lowest]-5 [highest]). Even though their motivation for participating in the program was not specific before the program, after the program, they reported having a more concrete image and specific form of what they learned from an international perspective. The participants enjoyed communication with others from diverse backgrounds while recognizing the difficulty of understanding different values. They also expressed satisfaction with their understanding of occupational therapy professionals and diverse societies, including medical systems from other countries.

**Conclusions:**

Even though the analyzed sample data were small, these findings suggest that the program in this study may provide the participants with valuable opportunities. The virtual exchange program could foster students to cultivate qualities such as problem-finding or problem-solving and having interactions with groups from diverse backgrounds.

## Introduction

### Background

As globalization has expanded, education for global human resources in medical education is increasingly being promoted. The significance of international exchange activities in professional fields beyond cultural exchange is also expected. In addition, there is a growing need in the rehabilitation profession to provide medical care to people with diverse backgrounds and collaborate with foreign medical professionals for the further development of the professional field. However, due to travel restrictions, international programs faced serious challenges during the COVID-2019 pandemic, and virtual learning was pursued as an alternative approach [1-3]. The objectives of international exchange programs include not only exchanging academic ideas but also gaining experience with interactive communication in different cultural contexts. Even when restrictions are placed on traveling abroad, educational institutions are expected to manage international academic and cultural programs by synchronizing online programs. During the COVID-19 pandemic, programs were conducted in which students joined from 2 or more universities and participated in activities together for several weeks [4], as well as a successful interactive program to learn professional knowledge and cultivate an international perspective [5,6]. As global health requires significant societal and pedagogical transformations regardless of physical mobility, virtual collaborative international learning has the potential to transform students in the health professions into global human resources. This approach can offer feasible, meaningful, and cost-effective solutions to students in the health professions, thereby enriching cultural competence and global understanding of health through virtual knowledge exchange [6,7].

However, in the present postpandemic era, the restarting of in-person educational programs that involve traveling abroad has created a new situation for virtual international exchange programs. The rapid development of IT during the COVID-19 pandemic has promoted learning knowledge that supports international collaboration toward addressing increasingly complex societal issues, and as such, higher education needs to leverage virtual education while addressing issues such as access, equity, cost, and ecology. In addition, virtual international education promotes internationalization at home (IaH) and provides more opportunities for students on campus to experience internationalization and develop an international orientation. IaH through virtual environments may provide benefits such as fostering a collaborative and diverse online community as a source of social and professional support and networking [8]. In globalization, virtual education may be a part of the essential skills and experiences necessary for generations familiar with and required to acquire IT skills and literacy. Students can feel more open to people from diverse backgrounds and international careers while becoming more familiar with different online technologies [8]. In the health professional education area, pharmacy students have learned about similarities and differences in socioeconomic determinants of health as well as the structure, functioning, and financing of different health care systems. Technology enables more students in diverse geographic locations to be exposed to various perspectives and health care experiences [9]. Student experiences of diversifying and further integrating using virtual platforms can help promote adaptation to global society, develop novel skills and knowledge, and contribute to future development in the medical field.

### Global Human Resource Development in Virtual Education

Effective virtual international education should not be considered a replacement for traditional exchange programs that include travel abroad, but rather, a different educational method for increasing international orientation and acquiring the global qualities needed for IaH. Beelen et al [10] describe IaH as the purposeful integration of international and intercultural aspects into students in their home countries. With improvements in IT, virtual collaborative international learning can be effectively managed for interactive communication. For instance, in 2006, the State University of New York developed a collaborative online international learning (COIL) program to engage in an international experience as a virtual type of exchange education based on a collaborative and social constructivist learning approach [11]. As interest in COIL increased, it was regarded as not only an alternative method, but also a method that considered the carbon footprint and environmental impact of physical mobility in regard to air travel [12]. The COIL program offers students an authentic learning experience at their home institution and helps them develop intercultural competencies while serving as a relatively environmentally friendly, sustainable method to internationalize the curriculum. COIL has several characteristics that facilitate learning effectiveness [12]: collaboration between two or more educators from different institutions of higher education in different countries, the co-design and cofacilitation of a joint collaborative course by educators, an emphasis on learning intercultural content and developing collaboration skills through collaborative learning assignments, and several synchronized online meeting times during the course. In the medical professional education field, some studies have been conducted through the COIL program [13,14]. For instance, through the COIL program, nursing students had meaningful, valued engagement with peers in another country to prepare students for diverse, multicultural work settings for their professional futures. The COIL program also helped faculty members conceptualize lessons that promoted intercultural respect and appreciation using online learning methods [13]. Even though English was a second language, the program allowed nursing students to increase their intercultural sensitivity, improve their English proficiency, and obtain more confidence in their interactions and communication with individuals with different cultural backgrounds [15]. Moreover, COIL experiences opened their eyes to a new way of thinking about preconceived ideas developed without actual knowledge of the situations outside their country, indicating that these cultural encounters led to better cultural awareness, humility, knowledge, skills, and desires [16].

Within virtual education, effectiveness should be judged in terms of not only understanding diverse cultures and increasing cultural awareness, but also developing global human resources. The concept of global human resources includes not only language and communication skills, but also initiative, positivity, a spirit of challenge, cooperativeness, and a sense of responsibility, as well as cross-cultural understanding, a broad range of education, deep expertise, problem-finding and -solving skills, teamwork and leadership, and a sense of ethics. These are not merely limited to an understanding of other cultures and language skills, but also correspond to the professionalism and qualities required of medical professionals. Therefore, cultivating global qualities contributes to not only internationalization but also professionalism in the development of students and institutions. In the medical area [17], the acquisition of necessary skill sets to foster globally competent dental students was enhanced through international virtual team-working, problem-solving, and person-centered multidisciplinary care planning activities. An online program fostering these qualities was beneficial for students from a broader global perspective and demonstrated an appreciation of the importance of delivering culturally sensitive person-centered dental care [17]. Furthermore, by combining virtual exchange and clinical simulation-based experiences for nursing students, the program was associated with statistically significant gains in the cultural intelligence of nursing students to function effectively in situations where cultural diversity was present, which was considered to have a positive impact on their future competence as global health care workers [18]. Another study found that nursing students reported gaining a profound comprehension of, and broadened perspective on, global health and cultural awareness, thereby enhancing their cultural competence [19]. Furthermore, such investments in international virtual education programs have the substantial benefit of offering all students on campus the opportunity to acquire global qualities. As a matter of equity, resources devoted to virtual exchange programs pay substantial dividends for students in historically marginalized and underresourced groups that have been underrepresented in international curricular experiences [20]. Therefore, well-organized virtual programs can be effective for various students to cultivate professional qualities as demonstrated by improvements in global cultural intelligence and academic performance. However, to our knowledge, no studies on rehabilitation professionals, including those in occupational therapy (OT), have been reported. Furthermore, no studies have been conducted on virtual international exchange programs between multiple non–English-speaking countries.

### Aim of This Study

The development of virtual international exchange programs during the COVID-19 pandemic as a new method of fostering global human resources should have led to new adaptations, innovations, and equity considerations. Therefore, such programs need to be examined in terms of their feasibility and sustainability, not just as alternative options for in-person programs. Given this background, this study aimed to examine the effectiveness of virtual international exchange programs for improving academic learning skills, professional knowledge, and global communication abilities in group interactions from student perspectives and suggest meaningful ways to promote global education (global health knowledge and cultural intelligence). This report also aims to contribute to the educational effectiveness of virtual international programs in the rehabilitation professional education area in the postpandemic era, as well as to the development of global education, including OT education, via virtual learning among rehabilitation professionals. In addition, this research can be expected to contribute to the development of global human resources in various health professions through the exchange of information from multiple countries and various international backgrounds.

## Methods

### Study Design

**Figure 1.**
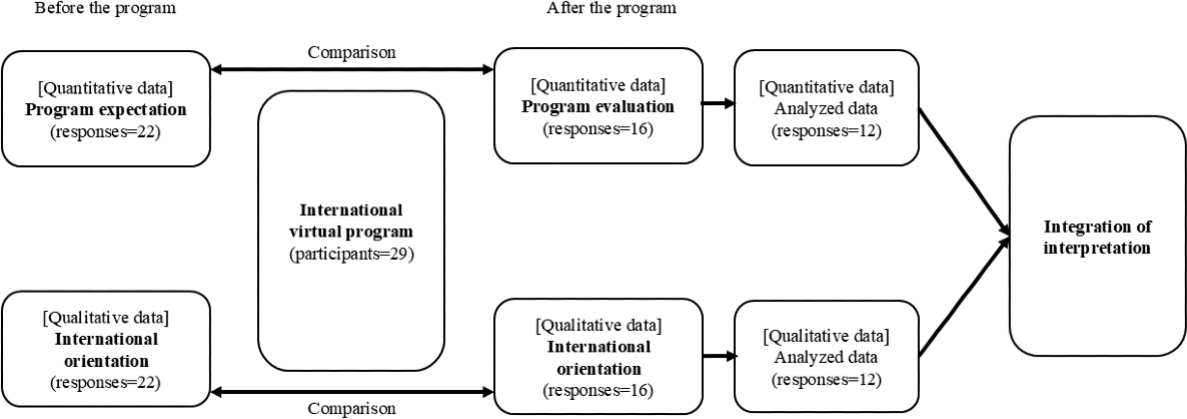
Study process.

This quasi-experimental study was conducted as intervention research using a before-and-after design and used a convergent parallel mixed methods approach (Figure 1) in which the researcher converges or merges quantitative and qualitative data to provide a comprehensive analysis [21]. In this study, both quantitative and qualitative data were collected before and after the intervention program and compared to evaluate students’ international attitudes and perspectives. After the analysis, the quantitative and qualitative data were integrated into the interpretation of the overall results.

### Ethical Considerations

The questionnaire and methodology for this study were approved by the Research Ethics Committee of Arakawa Campus Tokyo Metropolitan University (ethics approval number 23060) and registered in the UMIN-Clinical Trials registry system (UMIN trial number UMIN000050884, Reception number R000056060). This study was performed in accordance with the ethical standards laid down in the 1964 Declaration of Helsinki. Participation in this study was voluntary, and informed consent was obtained in writing from all participants before the study began. All participants’ data were collected as anonymized data, and participants joined the research with no rewards.

### Program Contents

The program was mainly organized by the host university (Tokyo Metropolitan University, Japan), with collaboration from 6 institutions from 4 countries (Angeles Foundation University, the Philippines; Bangladesh Health Profession Institute, Bangladesh; Chaing Mai University, Thailand; Mahidol University, Thailand; the Surakarta Ministry of Health Polytechnic, Indonesia; and the University of Indonesia, Indonesia). Tokyo Metropolitan University concluded a Memorandum of Understanding with all institutions to build relationships before the program was planned and primarily managed the program preparation through good collaboration. The goals of the program were to cultivate an international perspective, to understand OT in Asian countries through lectures and group work, and to learn basic research and presentation skills. During the development of the program, the Attention, Relevance, Confidence, and Satisfaction (ARCS) motivation model developed by Keller [22,23] was applied. The ARCS model proposes significant concepts regarding student attention in class, relevance to participants, student confidence in class, and student satisfaction. The ARCS model has been widely accepted and used to promote student motivation and active participation in class [24,25], and the online program was conducted effectively based on the ARCS model [5]. Hence, in the program, lecturers selected appropriate topics to achieve the program goals. In addition to the lecture contents, assignments and activities were organized to improve student understanding and maintain motivation in considering their academic progress and interests. All lecturers among the collaborative institutions involved in the management of the program agreed on the program contents.

As shown in Table 1, synchronized lectures from 4 countries were provided, and students were invited to join multimix group work on the last day. The group work topics included the role of OTs in the following areas: employment support in mental health, school, care for older adults, remote or isolated area care, assistive devices, stroke, addiction, and stigma. Students made 3 requests on the first day and were assigned to group work on the second. Lectures were provided by 4 countries, and students engaged in interactive group work within a small cultural program. A videoconferencing application (Zoom Communications, Inc) was used for the interactive live lectures, and a free cloud service (Google LLC) was used to share information and materials through an original website. The lecturers and students had access to the program contents and could provide or get handouts and recorded lecture materials for preparing and reviewing. During the program, students were encouraged to work together on their assignments and communicate with each other outside of the lectures through various tools and applications.

In all 7 institutions, the program participants were invited to participate in the virtual exchange program. In total, 30‐35 students (n=5 from each institution) were targeted to join the program. The students were able to choose to participate in the program regardless of their involvement with the research data collection.

**Table 1.** Program contents from February 27 to March 7, 2024.

Session^a^	Date	Contents
1	February 27, 2024	Orientation: medical and welfare system in Japan, long-term care insurance (by Tokyo Metropolitan University lecturer)
2	February 28, 2024	Health and welfare system in Thailand (by Chiang Mai University lecturer)
3	February 29, 2024	Community care in Indonesia (by Surakarta Ministry of Health Polytechnic lecturer)
4	March 4, 2024	Community care in Indonesia (by Bangladesh Health Professions Institute lecturer)
5	March 7, 2024	Students’ project presentations

a Each session was held at the following times: 10 AM–11:30 AM (Bangladesh), 11 AM-12:30 PM (Indonesia), 12 PM-1:30 PM (Philippines), 1 PM-2:30 PM (Japan).

### Participants

The research participants were recruited from an exchange program. The students received no credits because the program was optional. Regarding recruitment, after obtaining ethical approval, Tokyo Metropolitan University lecturers requested research collaboration with joint researchers from each institution or the head of the department via a request letter. After obtaining approval from each institution, a coresearcher (KI) asked participating students by email to complete a questionnaire survey.

### Data Collection

Questionnaires were prepared to examine the influence of virtual international learning and the students’ international orientation. In addition to age, sex, and school year, before joining the program (within 5 d) and after joining the program (within 5 d), the research participants were asked to complete an online questionnaire survey that consisted of 2 sections: program expectations evaluations and international attitude. The online survey was conducted in accordance with the CHERRIES (Checklist for Reporting Results of Internet E-Surveys).

#### Program Expectations or Evaluations

The questionnaire asked about the students’ expectations and evaluations based on the factors of academic experience, scientific learning skills, group interactions, learning value, program organization, assignments, and workload (refer to Multimedia Appendix 1). Responses were given on a 5-point Likert-type scale. These questionnaires on program expectations (before the program) and evaluations (after the program) were developed based on the student evaluation of educational quality questionnaire and other materials [26-28]. The student evaluation of educational quality questionnaire was originally developed by Marsh [26] as a student course evaluation in higher education and is now widely used around the world in multiple languages. The original questionnaire serves to evaluate the term course. In this study, we developed questionnaires that included items appropriate to the content of the program that could confirm the expected level of achievement. We selected questions that could compare the students’ expectations and satisfaction before and after the program. In addition, some expressions were adjusted to fit the program. In total, 15 questions were used before the program to ask about program expectations, and 30 after the program to ask about program evaluations and satisfaction.

#### International Orientation

This questionnaire (free comments) asked about student motivation to join the international exchange program, the influence of the experience of joining the program, and international attitudes (refer to Multimedia Appendix 1). The questions on international attitudes were developed based on the revised version of IP [29,30], which was developed by Yashima [29,30], who devised an evaluation method for international orientation, from the work of Gardner [31] and Dörnyei [32] on the relationship between foreign language acquisition and international orientation. In this study, we used the IP factors that were appropriate for this study, including intercultural approach (avoidance) tendency, interest in international vocation, ethnocentrism (reaction to different customs or values or behaviors), interest in foreign affairs, and having things to communicate (willingness to communicate to the world).

### Statistical Analysis

After the coresearcher (KI) removed all personally identifiable information, numerical data (questionnaire on program expectations or evaluations) were analyzed using descriptive statistics. Qualitative data (questionnaire on international orientation) were subjected to analysis using generative coding by means of steps for coding and theorization (SCAT), which is a qualitative analysis method developed based on the concept of grand theory [33,34]. This method is particularly effective for analyzing relatively small amounts of qualitative data, such as those from a single case or free comment sections of a questionnaire. SCAT consists of 4-step coding using a matrix accompanied by a procedure that describes the storyline and theory by spinning the constructs. The steps followed in SCAT are (1) a word or phrase in the data to focus on, (2) a phrase outside the data to paraphrase, (3) words and phrases to explain these, and (4) emerging themes and concepts. This 4-step coding process creates a storyline or theory that weaves together the themes or constitutive concepts in step 4. In particular, SCAT, as revised by Fukushi and Nago [34,35], is an effective method when there are many cases in which the linguistic data are very short (eg, 1 or 2 lines), such as descriptions in the free comments column of the collected questionnaires. In the revised procedure, the intercepted data are grouped, paraphrased, and conceptualized. In this study, we used the revised version of SCAT for the data analysis. The first author (NS) analyzed the data, the validity of which was then confirmed by one of the coauthors (KI), who had experience with qualitative research and over 20 years of experience in verifying medical education data, followed by the other 3 coauthors (SSS, MHS, and SM). All data were analyzed and interpreted based on the participants’ evaluations of the international program experience.

## Results

### Participant Characteristics

In total, 29 students (10 from Indonesia, 6 from the Philippines, 5 each from Japan and Bangladesh, and 3 from Thailand) joined the virtual exchange program from February 27 to March 7, 2024, among whom 22 completed a questionnaire before and 16 after the program. Data (from both before and after the program) were available from 12 participants (4 male and 8 female; mean 22.5, SD 2.6 years, age range 19‐28 years; response ratio: 41.4%) for analysis. Regarding the year of schooling, 3 participants were in their second year, 4 in their third, and 5 in their fourth (Figure 1)

### Quantitative Data: Program Expectations or Evaluations

Table 2 shows the results of the questionnaires conducted before and after the program. The level of achievement before and after the program was compared for 15 items. The results indicated that the students were satisfied after the program. The students had high expectations before the program, and overall, these expectations were met in terms of academic experience, scientific learning skills, and group interactions. The students responded that they gained valuable knowledge and ideas from the program. The program contents were evaluated as being well prepared, and almost all the participants wanted to join a similar program if given the opportunity. However, some students expressed the need for more support from lecturers to engage in the program and the need to establish a good transmission of internet. In addition, the program schedule was a bit tight because regular classes were being managed at the same time. Overall, the program contents and workload seemed to be reasonable for the students. As a result, they gained new experience in deep expertise, scientific learning through problem-finding and problem-solving, and group interactions in an international context. Although significant differences were not found between before and after the program, the results indicated that the program met the students’ high expectations.

**Table 2.** Results regarding student expectations and evaluations before and after the program (N=12).

Program expectation questionnaire (before program)	Student evaluation questionnaire (after program)	5-point Likert scale	Before program, mean (SD)	After program, mean (SD)
Academic experience		Strongly agree (5)-strongly disagree (1)		
I feel I will be able to increase my knowledge about OT^a^ field.	I increased my knowledge about OT field.		4.5 (0.90)	4.5 (0.67)
I feel I will be more interested in academic learning.	I developed my interests in academic learning further.		4.1 (1.00)	4.3 (0.89)
I expect my knowledge of academia will change.	My knowledge of academia has changed.		4.4 (0.67)	4.0 (0.95)
Scientific learning skills		Strongly agree (5)-strongly disagree (1)		
I expect to learn about collecting and searching information skills.	I learned collecting and searching information skills.		4.8 (0.45)	4.9 (0.67)
I expect to learn about presentation and discussion skills.	I learned presentation and discussion skills.		4.8 (0.45)	4.6 (0.79)
I feel preparing for the presentation assignment will be good training for me.	Preparation for the presentation assignment was good training for me.		4.7 (0.49)	4.6 (0.79)
Group interaction		Strongly agree (5)-strongly disagree (1)		
I feel I will be able to understand my friends’ thoughts in other countries.	I understood my friends’ thoughts in other countries.		4.5 (0.52)	4.6 (0.67)
I feel I can communicate with OT students and OT lecturers from other countries.	I communicated with OT students and OT lecturers from other countries.		4.5 (0.52)	4.6 (0.67)
I feel I can learn from each other.	I learned from each other.		4.6 (0.51)	4.7 (0.65)
I expect to cultivate an international perspective through a student exchange program.	I cultivated an international perspective through a student exchange program.		4.5 (0.67)	4.6 (0.79)
—^b^	The atmosphere was good for sharing my ideas and thoughts in group discussion.		—	4.3 (0.78)
Learning value		Strongly agree (5)-strongly disagree (1)		
I expect to find the program intellectually challenging and stimulating.	I have found the program intellectually challenging and stimulating.		4.4 (0.67)	4.3 (0.75)
—	I have learned something that I consider valuable.		—	4.6 (0.67)
I feel my interest in the subject will increase as a consequence of this program.	My interest in the subject has increased as a consequence of this program.		4.5 (0.67)	4.2 (0.83)
—	I have learned and understood the subject materials of this program.		—	4.7 (0.65)
Organization		Strongly agree (5)-strongly disagree (1)		
—	The lectures given by lecturers were very clear.		—	4.4 (0.79)
—	Program materials were well prepared and carefully explained.		—	4.5 (1.00)
I expect to deepen my knowledge about the areas of interest, and I will learn at my own pace through the program.	I learned interesting areas deeply at my own pace through the program.		4.4 (0.67)	4.2 (0.83)
—	The amount of support provided by lecturers was sufficient during the program.		—	3.9 (1.00)
—	Internet environment and technical support for the use of device and application were sufficient during the program.		—	3.9 (0.90)
Workload or difficulty		Too easy (5)-too difficult (1）		
—	How is the program difficulty?		—	3.3 (0.75)
—	How was the assignment workload?		—	3.1 (0.79)
—	How was the program pace?		—	2.6 (0.67)
I think the level of program content is suitable for me.	Overall, from Q23-25, the level of program content was suitable for me.	Strongly agree (5)-strongly disagree (1)	4.1 (0.67)	4.2 (0.72)
Assignment		Strongly agree (5)-strongly disagree (1)		
—	The required reading materials were valuable.		—	4.4 (1.00)
—	Required assignments contributed to appreciation and understanding of OT in the domestic and international fields.		—	4.6 (0.67)
Others		Strongly agree (5)-strongly disagree (1)		
—	Do you want to join a similar program if you have another opportunity?		—	4.7 (0.65)
—	The date and time schedule were reasonably good.		—	3.8 (1.19)
—	I actively participated.		—	4.3 (0.75)
How much do you expect out of this program overall?	The program met my expectation.	Very much (5)-not at all (1)	4.1 (0.90)	4.3 (0.75)

a OT: occupational therapy.

b Not applicable.

### Qualitative Data: International Orientation

As a result of the SCAT coding of data before the program, 20 group concepts and 6 overall concepts emerged regarding international orientation (Table 3). In the text, [ ] indicate overall concepts and < > indicate group concepts.

Students decided to join the program because they were interested in [Learning new knowledge in the professional area] to <expect to know and learn about OT in other countries> and, they were interested in the [opportunity to have international experiences] and <seeking that opportunity>, but sometimes they had <no opportunity to exchange with people from other countries> or <they did not use that actively and usually>, even though they had the opportunity to communicate with people from other countries before joining the program. In addition, they were <interested in studying or working abroad>, but it might be <difficult for some of them due to financial and family concerns>. The virtual program was not concerned about these issues; therefore, it was easy to join to have international experiences. Furthermore, the students expected to [Have new international experiences with different cultures and values] from <making friends and having interactive communication with students in other countries> and <learning and experiencing something new>. In the international context, they <expected the program to lead to being more open-minded internationally>, as well as <learning about the cultures and values of other countries would make me change something> and being <interesting and fun to learn about diverse cultures and values>. On the other hand, some students were worried about <difficulties in understanding other cultures and values>. Similarly, in [international interactive communication], some students had <expectations of and interests in exchanging ideas and opinions>, <expectations of multicountry exchange experiences>, and <using English as a second language>; however, others experienced the <challenge of exchanging ideas and opinions>. Moreover, regarding [general global attitude sensitivity], depending on the individual, some <cared about international issues as usual interest>, but others did not or had <no opportunity to learn about that information>. They were interested in other values or study areas in other countries, but their general international awareness did not always explain their motivation to join the program.

**Table 3.** Results regarding concepts, group concepts, and participant descriptions of international orientation before the program (N=12).

Group concepts	Examples of participant descriptions
Concept: [Learning new knowledge in the professional area]	
Expectation to know and learn about OT^a^ in other countries	Good opportunity to know more about different dimensions of OT in different countries. I want to know about the OT field in other countries.
Expectation to experience an international perspective in the medical health professional field	From lectures given by other countries, I can compare their system with ours. In the discussion session, my understanding about health care will be deepened from a student perspective. I think this is a great opportunity to get a glimpse of the wider global professional perspective regarding OT.
Concept: [Having new international experiences with different cultures and values]	
Hope to make friends and have interactive communication with students in other countries	I would like to participate in discussions with other students from other countries. Participating in the virtual exchange program will improve my communication skills.
Expectation of learning and experiencing something new	Sounds interesting, I will learn a lot from the program. It can increase my vision about how I can become successful in the future.
Expectation of the program leading to a more open-minded international perspective	It drives me to behave as politely as I can to accept diversity. I will learn more about other perspectives, and it will also affect my international perspective.
Interesting and fun to learn about diverse cultures and values	It is very fun to discover the diverse values and ideas of others. I really enjoy and am open-minded about interacting with and discussing things with people who have different values and ideas. I am very interested in learning about culture and other things from abroad.
Perceiving difficulties in understanding other cultures and values	Sometimes it is hard to be on the other side of a position. I feel like it takes effort to understand fully people from other cultures with different opinions.
Learning about other cultures and values changes me	It encourages me to dive into other perspectives affected by my culture. Exchange programs can open my mind and teach me lessons.
Concept: [Opportunity to have international experiences]	
Interested in or aspiration of studying or working abroad	I have a big dream to study overseas. I am very motivated to participate in any cross-cultural opportunity. I am really interested in studying abroad and later working abroad to increase my knowledge and experience. I really want to continue my OT study abroad.
Difficulties of studying and working abroad	I know that I will face many challenges abroad. I have financial concerns.
Seeking more opportunities to communicate with people from other countries	If I have an opportunity to contact people with other nationalities, I will be grateful. I would like to have multicultural exchanges.
No previous exchange opportunities with people from other countries	I am a little hesitant. I have not had the opportunity to explore cultures in other countries.
Concept: [General global attitude sensitivity]	
Not interested in international issues or situations	I rarely consume news from other countries. I am not concerned about world affairs.
Interested in international issues and situations	We should know what is going on in other countries, this is our responsibility as human beings. I always follow international news because I think everything that happens always affects everyone. I am always curious about social conditions in other countries. I can learn from updated information related to policy, health, and so on in other countries.
Not enough opportunities to know about international issues or situations	I am not very informed about issues in other countries. I don’t have enough knowledge about international issues.
Concept: [International interactive communication]	
Expectations of and interest in exchanging ideas and opinions	I would like to express my opinions on diverse positions. I would like to share and discuss my perspectives with others. I would like to exchange and receive opinions from different perspectives as it would help me to reflect and modify my knowledge or viewpoints.
Challenge of exchanging ideas and opinions	I think it is challenging for me to exchange my ideas with others. I feel like it will take effort to organize and tell people with other nationalities to understand ideas fully because of language barriers.
Expectation of a multicountry exchange experience	I have the chance to work on projects and communicate with people from many countries. We will share our perspectives in groups with students from different countries.
Using English as a second language	It is a good opportunity to practice English skills. The language barrier creates some difficulties while communicating with people from other countries.
Concept: [Motivation to engage in international experience through external encouragement]	
Recommendations from others	Participating with some international OT students and teachers motivates me to learn more.

a OT: occupational therapy.

As a result of the SCAT coding of data after the program, 19 group concepts under 7 overall concepts regarding the participants’ international orientation were extracted (Table 4). In the text, [ ] indicate overall concepts and <>indicate group concepts.

Through the program, students reported [knowing and learning new knowledge about OT and other topics in other countries in an international context]. They <learned about OT, including clinical and working situations in other countries> and <gained a wide knowledge of other countries>. With this [opportunity to have international experiences], they <enjoyed it by having international exchange experiences> and <multicultural exchange opportunities>. Some were <seeking the next opportunity for an international exchange experience> and were <interested in or aspiring to study or work abroad>, but they had <difficulties studying or working abroad> and did not have <much opportunity for international exchange after the program>. In the program, the students had the opportunity to [understand the cultures and values of others], which was good in terms of <learning from others and enjoying diverse values and ideas>, but it was <not easy to understand others> in some situations. The program involving multiple countries helped promote [mutual understanding in an international context] in terms of <understanding the viewpoints of others in an international context>, <knowing international perspectives>, and <gaining new ideas from others>. The students reported <enjoying sharing opinions, ideas, and interests with others> in [interactive communication with others], even though one student perceived a <language barrier>. After the program, some students reported the need <to know more about international issues and situations> and expressed <interest in and care about international issues and situations>, but others did <not care about international issues or situations> as part of [general global attitude sensitivity]. They had the experience of learning about the knowledge and values of other countries and enjoyed having interactive communication with people from other cultures and backgrounds.

The qualitative questionnaire was conducted from the perspective of intercultural approach tendencies; interest in international vocations; reactions to different customs, values, or behaviors; interest in foreign affairs; and having things to communicate; these corresponded with the group concepts. Regarding the comparison of data between before and after the program, before joining the program, participants were motivated by interests and curiosity about knowing about OT from overseas, not the concrete contents. However, after the program, they had more concrete images about specific forms (eg, employment support and clinical skills) from what they learned from an international perspective. A lot of the participants sought opportunities for international exchange, but could not easily find them. Therefore, the chance to join the program was valuable for students, who reported that they wanted to participate the next time they had the opportunity. However, no participants indicated that they wanted to expand opportunities by themselves in the future. Some participants said that they would like to study or work abroad, but felt that it was difficult because of financial and family issues. As for attitudes toward different cultures and behaviors, the participants attempted to understand and enjoyed communicating with others from diverse backgrounds, even though they recognized that understanding different values was not always easy. On the other hand, interest in general international affairs was not necessarily a motivation for participation, and no changes were seen as a result of participating in the program.

**Table 4.** Results regarding concepts, group concepts, and participant descriptions of international orientation after the program (N=12).

Group concepts	Examples of participant descriptions
Concept: [Knowing and learning new knowledge about OT^a^ and other topics in other countries in an international context]	
Learning about OT, including clinical and working situations in other countries	We talked about the details of how OTs work and the OT process in every country. I did not know anything about the system before. Now I know about OT situations in different countries. I have learned so much about OT situations in other countries.
Gaining wide knowledge about other countries	My knowledge horizons were widened with OT students from other countries. I got to know things I did not know before.
Concept: [Understanding other cultures and values]	
Not easy to understand others	I feel that it takes effort to understand and adapt to other people from different countries.
Learning from others and enjoying diverse values and ideas	I really enjoyed hearing about and discussing differences in values and cultures. I enjoy interacting with people who have different ideas. I find it interesting that I can have discussions with students from other countries and see their perspectives.
Concept: [Mutual understanding in an international context]	
Understanding the viewpoints of others in an international context	I gained a deep understanding about the different perspectives of other countries compared with us. It allowed me to respect the other participants. I know for a fact that no country has a perfect program, each has its own flaws and we can continue to grow by learning and appreciating them.
Knowing international perspectives	It gave me a lot of new information about other countries so I can learn and know more about international situations. I do not have that much knowledge about the OT field in other countries, so the program affected my international perspective. My international perspective did not change.
Gaining new ideas from others	I was able to improve my knowledge and see other perspectives. I love to learn about how people think differently.
Concept: [Opportunity to have international experiences]	
Having and enjoying opportunities to have international exchange experiences	I enjoy having the ability to communicate and participate in an international program. This was the first time I had an experience like this.
Seeking new international exchange experience opportunities	I would like to if there is another opportunity. It was wonderful and I hope to participate in more programs like this if I get the chance in the future.
Multicultural exchange opportunities	I would love to have the opportunity to communicate with students from other nationalities and cultures. I really like multicultural exchanges. I was usually a little hesitant to contact strangers, but now I am happy because I can contact students with other nationalities.
No opportunity for international exchange after the program	I very rarely have this opportunity. We didn’t have any international program to facilitate us.
Interested in or aspiring to study or work abroad	I always dream about working outside the country. I had a dream to pursue higher education abroad. My goal is to continue my studies abroad. I hope I have the chance someday.
Difficulties studying or working abroad	I have financial concerns. I have some family concerns. Working abroad is equally exciting and challenging.
Concept: [General global attitude sensitivity]	
Not interested in international issues or situations	No time to see. Normally I do not care about what is going on.
Need to know more about international issues and situations	I think I need to have a look at situations happening around the world. I am interested in other countries because I can compare and learn things from them. I should be interested in it because it is called globalization.
Interested in and care about international issues and situations	I am really interested as it gives me the opportunity to gain information about the world, which is very important in this era of digitalization. Now, I am interested. It is a very helpful example for us to develop.
Concept: [Interactive communication with others]	
Enjoying sharing opinions, ideas, and interests with others	I really enjoy sharing my thoughts and interacting with people from other countries. I really like to express my opinions on diverse positions. I felt like they were close to me.
Language barriers	It was a bit difficult to communicate with others while we were using our second language.
Concept: [Difficulty arranging the schedule of the international program as an optional program]	
Time management	I did not have time to participate in our class.

a OT: occupational therapy.

## Discussion

### Principal Findings

This study examined the educational effectiveness of a virtual international exchange program focusing on fostering global qualities involving academic and professional learning and international orientation from students’ perspectives. The research participants expressed satisfaction with the program in regard to cultivating an international perspective, understanding OT professionals from other countries, learning academic skills through international communication, and learning about diverse cultures and societies, including medical systems. Even though the results of the quantitative survey did not indicate significant differences, the program participants had high expectations and indicated that they were satisfied with the contents. In addition, the program gave participants a valuable opportunity to cultivate global qualities such as problem-finding and problem-solving, and to have group interactions with people from diverse backgrounds.

The results could indicate that the virtual program was capable of meeting student expectations for international experience through the learning of professional knowledge and communication skills, in line with previous studies [6,7]. This program consisted of lectures, understanding medical situations in other countries through OT professional knowledge, and interactive group work followed by presentations. This program was developed to maintain student motivation and cultivate global qualities based on the ARCS model. The participating students expected to gain new knowledge and ideas about professional areas in international contexts and communicate with others with diverse opinions and backgrounds; from this perspective, their expectations were met. Even though the results of the quantitative survey did not show significant differences between before and after the program because of the participants’ high expectations (ceiling effect), the results indicated a mutual understanding of diverse opinions and a recognition that the participants’ differences and similarities were valuable. In addition, the vague image of international exchange before the program became more concrete and practical after the program. Thus, the participants had the opportunity to foster their international orientation. On the other hand, the program period may have affected workload or difficulty; for example, the program pace was a bit fast, so the student might not have had time to absorb and review knowledge or brainstorm with international friends in their groups before presenting an assignment. However, the schedule for the long-term program is difficult to structure because of the busy curriculum, including practical placement in health education in multiple institutions. Therefore, even in the short term, the program should make more improvements from the students’ perspective.

### Implications of the Findings

The virtual program in this study provided students with meaningful experiences, in terms of cultural awareness, competence, and intelligence, for growing as global human resources, in line with previous studies [13,16,19]. Participants showed interest in international communication with professional knowledge both before and after the program, including having friends with different values and learning new things about other countries, even though some of them felt difficulties in communicating with those with different backgrounds. After the program, students reported having more concrete knowledge about OT in other countries and communicating with people with various values through professional knowledge. This may indicate the need to cultivate the ability to build a global community with a common understanding of global health and raise awareness of safety and cultural competency for people from diverse backgrounds in health care. Global institutional collaboration fosters international collaboration between not only students but also educators and researchers [19]. In addition, the virtual interactive program could enhance the development of student competency by contextualizing knowledge, fostering collaboration and innovation among universities, creating an international professional network for students and instructors, and promoting professional skills [36].

Therefore, it is essential to manage communication in virtual programs and foster solid relationships among educational institutions. Student experiences could lead to a global campus environment, which includes faculty and administrators. The establishment of virtual programs relies on existing relationships, clear communication, and a commitment to collaborate [37]. In addition, communication within each institution and with participating students is key to a successful program. Some participants reported that they might have needed more support from faculty members. Interuniversity communication and collaboration among administrators, faculty, prospective students, and partner universities are also important [36,37]. Furthermore, a previous study demonstrated that in practice, these strategies need significant modifications, at least in part, to suit local contexts. Regarding logistical support to achieve effective internationalization “at home,” the present program, which included participants from 5 different countries, should have given more consideration to mutual understanding [38]. Other concerns in regard to the management of virtual programs include the online environment and program schedules [1,5]. In this study, the satisfaction score was not bad, but the satisfaction level was lower than the other items. Therefore, communication among institutions to manage and arrange schedules is important, and a solid, reliable relationship is essential to improve virtual educational programs.

Due to its short duration and contents, the program did not lead to significant changes in global attitudes; however, participants seeking an opportunity were able to learn and think about different values and conditions in other countries. As some students expressed concerns about financial and family issues in terms of studying or working abroad, virtual education was an easy option that allowed them to cultivate global qualities at home [8,20]. Furthermore, IaH programs based on virtual exchange and simulation have been shown to improve general student self-efficacy in the short term [39]. Virtual programs such as the one described in this study could therefore help foster an awareness of international orientation in global human resources. In OT education, OT knowledge and skills are mainly required; however, the experience of learning about and understanding different cultures and values is very helpful to support the lives of others and contribute to development in this field through collaborative communication with other countries [40]. Regarding virtual environments, even though students have adequate communication tools, interactive communication can be difficult, and thus, facilitating interactive activities such as group work is needed [6]. In this study, the participants expressed satisfaction with interactive communication, even in English as a second language. Therefore, the COIL program helps students cultivate meaningful interactive communication skills as a global quality to develop and coordinate team management skills among individuals with various values.

In this postpandemic era, some students may regard virtual education as an alternative option to in-person learning by traveling abroad. However, educators need to demonstrate the value and benefits of virtual education as IaH and clarify how virtual learning abroad programs should be promoted to students [41]. It was clear from the literature that international virtual education can not only maintain and create sustainable ties with international partners, which adds depth and richness, but also provides opportunities to create meaningful, lasting collaborative spaces for the ongoing expansion of global activities [42]. In addition, this type of program can be a positive influence as it gives all students on campus the opportunity to participate in global human resource development, even though the result of this study might indicate only the possibility due to the small sample size.

### Limitations and Future Research

This study has some limitations. First, the sample size was small, and the program was only conducted over a short period (1 wk). Therefore, the results may not fully reflect the students’ perspectives. On the other hand, conducting a virtual program with a much larger number of participants would make its quality difficult to maintain. Furthermore, at the moment, international programs are not mandatory in medical education in general, so it remains difficult to recruit many participants for practical reasons. Second, there is no comparison group in this study; therefore, the degree to which this program influenced student perspectives remains unclear. In the future, the effectiveness of the virtual program in each country should be assessed, and the program structure and preparation should be tailored to each country’s situation. This should lead to richer research data.

### Conclusions

As globalization has been increasing, it is important to provide medical care to people with diverse backgrounds and collaborate with foreign medical professionals for the development of the professional field. Effective virtual international education should not replace traditional exchange programs, but rather, offer a different educational method to increase international orientation and acquire global qualities as IaH. Within virtual education, effectiveness should be judged based not only on understanding diverse cultures and increasing cultural awareness, but also on developing global human resources. This study aimed to develop a meaningful virtual international exchange program to promote global education and examine academic learning skills, professional knowledge, and global communication ability from student perspectives. The results would be expected to contribute to education for the development of global human resources in the health professions through the exchange of information by people from various countries and international backgrounds. The present virtual international program was conducted using a quasi-experimental before-and-after design that used a convergent parallel mixed methods approach among 5 countries (Bangladesh, Indonesia, Japan, the Philippines, and Thailand). The program could provide students with meaningful experiences as global human resources in terms of cultural awareness, competence, and intelligence. In this postpandemic era, some students may regard virtual education as an alternative to in-person learning combined with traveling abroad. However, educators need to show the benefits and value of virtual education as IaH and clarify how these benefits should be promoted to students. Assigning projects that necessitate teamwork across different countries can compel students to engage more deeply with their peers, thereby fostering stronger communication skills and a better understanding of diverse perspectives. These collaborative tasks can simulate real-world scenarios where multidisciplinary and multicultural teams work together to solve complex problems. This approach can not only enhance learning experiences but also prepare students for professional environments where international collaboration is often essential. By requiring students to navigate language barriers, cultural differences, and varied working styles, such tasks can significantly enhance their global competence and teamwork abilities. These deeper interactions could bridge the gap between mere exposure to international elements and the development of a truly global perspective.

## Supplementary material

10.2196/77564 Multimedia Appendix 1 Questionnaires for before and after joining the program.

## Abbreviations

ARCS: attention, relevance, confidence, and satisfaction
CHERRIES: Checklist for Reporting Results of Internet E-Surveys
COIL: collaborative online international learning
IaH: internationalization at home
OT: occupational therapy
SCAT: steps for coding and theorization
