# Supplementary material for: Educational Effectiveness of a 5-Country Virtual Exchange Program for Internationalization in Occupational Therapy Education: Mixed Methods Study
Source: JMIR Med Educ. 2025 Nov 6;11:e77564. doi: 10.2196/77564 (PMC12591557; doi:10.2196/77564)
Supplement: Multimedia Appendix 1 [file mededu-v11-e77564-s001.docx]

**Questionnaire before joining the program**

The following questions are asking about your program expectations. Please select the most appropriate one of your current situations.

1．I feel I will be able to increase my knowledge about OT field.

2．I feel I will be more interested in academic learning.

3．I expect my knowledge of academia will change.

4．I expect to learn about collecting and searching information skills.

5．I expect to learn about presentation and discussion skills.

6．I feel preparing for the presentation assignment will be good training for me.

7．I feel I will be able to understand my friends’ thoughts in other countries.

8．I feel I can communicate with OT students and OT lecturers from other countries.

9．I feel I can learn from each other.

10．I expect to cultivate an international perspective through a student exchange program.

11．I expect to find the program intellectually challenging and stimulating.

12. I feel my interest in the subject will increase as a consequence of this program

13. I expect to deepen my knowledge about the areas of interest, and I will learn at my own pace through the program.

14．I think the level of program content is suitable for me.

15．How much do you expect out of this program overall?

The following questions are asking about your international perspective. Please describe your current situation in as much detail as possible.

1．What made or motivated you to participate in this program?

2．Is participating in an international exchange program likely to affect your international perspective? Why?

3．How do you usually think about having opportunities to contact other nationalities and cultures at school and in the community? (For example: "I would like to have multicultural exchanges, but I haven't had the opportunity." "I would like to, but I am a little hesitant.")

4．How do you think about studying abroad, working abroad, or volunteering abroad in the future? (For example: "I want to study abroad but have financial concerns," "My goal is to work abroad.")

5．How do you think about interacting with people who have different values and ideas? (For example: "I would like to be exposed to diverse values" or "I feel that it takes effort to understand people with different values.")

6．Are you interested in news and social conditions in other countries? Why? (For example: "I think I should be interested in it because it is called globalization" or "I don't care much about news and other affairs that don't directly concern me.")

7．How do you feel about having your own opinions from an international perspective and exchanging views with groups and individuals from other countries and multicultural backgrounds? (For example: "I would like to speak my opinion to diverse positions." or "I find it difficult to organize my opinion.")

**Questionnaire after joining the program**

The following questions are asking participants to evaluate the program. Please select the most appropriate one of your current situations.

1． I have found the program intellectually challenging and stimulating.

2． I have learned something that I consider valuable.

3．My interest in the subject has increased as a consequence of this program.

4．I have learned and understood the subject materials of this program.

5．The lectures given by lecturers were very clear.

6．Program materials were well prepared and carefully explained.

7．I learned interesting areas deeply at my own pace through the program.

8．The amount of support provided by lecturers was sufficient during the program.

9．Internet environment and technical support for the use of device and application were sufficient during the program.

10．I learned collecting and searching information skills.

11．I learned presentation and discussion skills.

12. Preparation for the presentation assignment was good training for me.

13. I increased my knowledge about OT field.

14．I developed my interests in academic learning further.

15．My knowledge of academia has changed.

16．I understood my friends’ thoughts in other countries.

17．I communicated with OT students and OT lecturers from other countries.

18．I learned from each other.

19．I cultivated an international perspective through students exchange program.

20．The atmosphere was good for sharing my ideas and thoughts in group discussion.

21．The required reading materials were valuable.

22．Required assignments contributed to appreciation and understanding of OT in the domestic and international fields.

23．How is the program difficulty?

24．How was the assignment workload?

25．How was the program pace?

26. Overall, from Q23-25, the level of program content was suitable for me.

27．Do you want to join a similar program if you have another opportunity?

28．The date and time schedule was reasonably good.

29．I actively participated.

30．The program met my expectation.

The following questions are asking about your international perspective. Please describe your current situation in as much detail as possible.

1．Was the program what you expected in this time? Why?

2．Has participating in an international exchange program affected your international perspective? Why?

3．How do you usually think about having opportunities to contact other nationalities and cultures at school and in the community? (For example: "I would like to have multicultural exchanges, but I haven't had the opportunity." "I would like to, but I am a little hesitant.")

4．How do you think about studying abroad, working abroad, or volunteering abroad in the future? (For example: "I want to study abroad but have financial concerns," "My goal is to work abroad.")

5．How do you think about interacting with people who have different values and ideas? (For example: "I would like to be exposed to diverse values" or "I feel that it takes effort to understand people with different values.")

6．Are you interested in the news and social conditions in other countries? Why? (For example: "I think I should be interested in it because it is called globalization" or "I don't care much about news and other affairs that don't directly concern me.")

7．How do you feel about having your own opinions from an international perspective and exchanging views with groups and individuals from other countries and multicultural backgrounds? (For example: "I would like to speak my opinion to diverse positions" or "I find it difficult to organize my opinion")

“This is a Multimedia Appendix to a full manuscript published in JMIR Medical Education. For full copyright and citation information see https://doi.org/10.2196/77564”
